# Advancing psychosocial care in cancer patients

**DOI:** 10.12688/f1000research.11902.1

**Published:** 2017-12-04

**Authors:** Luigi Grassi, David Spiegel, Michelle Riba

**Affiliations:** 1 University Hospital Psychiatric Unit, Integrated Department of Mental Health and Addictive Behavior, S. Anna University Hospital and Health Trust, Ferrara, Italy; 2 Institute of Psychiatry, Department of Biomedical and Specialty Surgical Sciences, University of Ferrara, Ferrara, Italy; 3 Department of Psychiatry & Behavioral Sciences, Stanford University School of Medicine, Stanford, California, USA; 4 University of Michigan Comprehensive Cancer Center, Ann Arbor, Michigan, USA; 5 Department of Psychiatry, University of Michigan, Ann Arbor, Michigan, USA

**Keywords:** psycho-oncology, cancer, psychological screening assessment, distress, psychopathology, psychotherapy

## Abstract

Cancer is a devastating disease causing significant psychological problems among patients and their families. In the past few decades, there have been growing implementation and dissemination of screening methods for the psychological consequences of cancer, including distress, depression, anxiety, post-traumatic stress, and demoralisation. Also, guidelines for the management of psychological distress have been developed and endorsed by a number of scientific cancer associations. This review examines some of the most significant related issues, also focusing on recent advances in psychosocial and psychopharmacological interventions as a part of a mandatory, integrated, and comprehensive approach to cancer care.

## Introduction

World Health Organization (WHO) projections estimate the incidence of cancer to increase exponentially by the year 2030, with the annual number of new cases rising from 14.1 million in 2012 to 21.6 million in 2030 and deaths due to cancer rising from 8.8 million worldwide in 2015 to more than 12 million in 2030 ^1^. At the same time, earlier diagnoses and improvement in cancer therapies have also led to an increase in survival that includes more than 300 million cancer survivors around the world. A broad implication of these figures involves the psychosocial impact of the disease, including emotional consequences, supportive care needs, and quality of life of cancer patients and their families. It is a fact that cancer is not only a series of very different diseases needing complex and multidisciplinary treatment but also a very stressful event with significant psychosocial implications related to physical, emotional, spiritual, and interpersonal dimensions. All aspects of life, including the parameters of time (e.g. the past, the present, and the future), space (e.g. one’s own individual space, one’s own home, and one’s own world context), and existence (e.g. confrontation with mortality) are altered by the diagnosis and treatment, recovery and long-survivorship, recurrence, or transition to palliative and end-of-life care.

In this review, we examine and summarise the recent data relative to the psychosocial consequences of cancer and the most significant policies regarding screening, assessment, and psychological treatment of emotional distress and concomitant psychosocial disorders among cancer patients.

## Psychological aspects of cancer

Recent data have examined the psychological consequences of cancer, showing that, at the physical level, cancer and cancer treatment have evident repercussions for body image, with differences between “visible cancers” (e.g. breast cancer and head and neck cancers) and “less-visible cancers” (e.g. leukaemia and lung cancer). The type of therapy, including surgery, chemotherapy, radiotherapy, immune therapy, and hormone therapy, also has important effects because of possible physical changes that may result (e.g. amputations, stomas, and hair loss) and symptoms (e.g. pain, nausea and vomiting, and fatigue) ^2^.

Declines in performance status and functional activity, problems in carrying on one’s own daily activities, poor concentration, memory impairment, or altered sexuality are important in influencing the psychological response of cancer patients ^3^. The loss of certainty, the instability of one’s own emotional status (e.g. fears, anxiety, worries, and sadness), the need to depend on others, the reduction of self-esteem, the change of perspective about the future, and the threat of possible death are some examples of the multitude of emotional effects and experiences cancer patients have to deal with during the trajectory of the illness. Spiritually, the whole set of personal values, the meaning given to one’s own life and existence, and the change in perception of time and being are important concomitants of cancer. The social and interpersonal dimension is also touched by cancer and its treatment. The sense of belonging (“to be with”) and communicating (“to have commonality with someone”) in the family, in the microcosm of close relationships, and in the macrocosm of broader work, social, and community involvement is also threatened or impaired by cancer. Feelings of loneliness and abandonment, problems in returning to work, marginalisation, or even stigmatisation are common issues that cancer patients report ^2, 3^.

All of these aspects may be evident in different phases of the trajectory of illness from diagnosis to survivorship or palliative care and the course of cancer (e.g. remission, recurrence, progression, and end of life). Cancer patients may adopt several styles of emotional, cognitive, and behavioural response to the disease. It has been shown that some styles, such as a fighting spirit (i.e. the tendency to confront and actively face the illness), seem to favour adjustment to illness, while others, such as hopelessness (i.e. the tendency to adopt a pessimistic attitude to the illness), anxious preoccupation (i.e. the tendency to constantly perceive the impact of illness in one’s own life), and denial, tend to be associated with poorer adjustment to illness and a higher risk of distress ^4^. Distress, in fact, is quite common in cancer patients and has been particularly studied over the last 20 years. It has been very broadly defined as “a multifactorial, unpleasant, emotional experience of a psychological (cognitive, behavioural, emotional), social and/or spiritual nature that may interfere with the ability to cope effectively with cancer, its physical symptoms and its treatment and that extends along a continuum, ranging from common normal feelings of vulnerability, sadness and fears to problems that can become disabling, such as depression, anxiety, panic, social isolation and existential and spiritual crisis” ^5^. Distress has also been termed the “Sixth Vital Sign” in cancer care ^6, 7^, and routine assessment and treatment of distress has been mandated by numerous regulatory bodies, including the International Psycho-Oncology Society (IPOS) Standard on Quality Cancer Care, stating that quality cancer care must integrate the psychosocial domain into routine care and that emotional distress should be measured as the sixth vital sign ^8^.

Many cancer patients have problems in adjusting during the illness trajectory, with “normal” psychological responses (e.g. sadness and preoccupation) shifting to more clinically significant states characterised by symptoms of anxiety or depressive disorders or other psychopathological conditions. Several factors have been enumerated as increasing the risk for the onset of psychopathological conditions, such as previous history of psychiatric disorders or trauma, inadequate and poor social support, uncontrolled symptoms, and female gender ^9^.

A psychopathological condition can be diagnosed, according to recent data in the literature, in 25–30% of patients by using the usual nosological systems of psychiatric classification (e.g. the International Classification of Disease, tenth edition [ICD-10] of WHO or the Diagnostic and Statistical Manual of Mental Disorders, Fifth Edition [DSM-5] of the American Psychiatric Association) ^10^. The most common diagnoses include stress-related and adjustment disorders ^11^, depressive spectrum disorders (including major depression and minor depression) ^12^, ^13^, anxiety disorders ^14^, and sexual disorders (e.g. loss of libido and anorgasmia) ^15^. Other psychological maladjustment conditions, such as demoralisation syndrome, health anxiety, and somatic symptom disorders, have also been underlined as clinically significant areas to be taken into account in cancer care ^16– 19^. Different phases of the illness trajectory can trigger emotional distress and the onset of psychological disorders, as reported in the US National Comprehensive Cancer Network (NCCN) distress management guidelines ( Table 1) ^9^.

**Table 1.** Some of the phases of cancer presenting risk for the onset of psychological distress (from National Comprehensive Cancer Network).

• Finding a suspicious symptom • Being informed about the diagnosis • Awaiting treatment • Change or end of treatment • Discharge from hospital • Surviving cancer • Failure of treatment • Recurrence or progression of disease • Advanced phase of illness • Approaching the end of life

Given the high number of cancer patients surviving their disease, survivorship has recently occupied more attention of both researchers and clinicians interested in psychosocial oncology, with data indicating that the creation and implementation of optimal methods for promoting the health and wellbeing of post-treatment survivors are critical for complete cancer care. The risk is that, since the disease has been treated and the patient recovered, psychological problems are not considered significant anymore. For example, one study indicated that only 40.2% of survivors reported having had a discussion with their clinicians about how cancer may have affected their emotions or relationships and that more than 90% of the barriers to the use of professional counselling or support groups identified by survivors involved lack of knowledge about, or perceived unavailability of, services ^20^. Thus, it is extremely important that clinicians monitor supportive care needs in patients surviving cancer, especially for those reporting fear of cancer recurrence, and anxiety, important issues present in cancer survivors ^21^. It is true that, in general, the rate of psychological morbidity tends to be lower in survivors rather than patients with active or recurrent disease ^22^. However, a series of problems can be present in this population (e.g. fear of cancer recurrence, possible long-term mental and physical side effects, economic burdens, and distress) ^23, 24^, including particularly those who received a cancer diagnosis during adolescence or young adulthood ^25^. With respect to this, Aaronson *et al*. ^26^ have underscored a series of psychosocial issues to be taken into account in a person-centered approach to survivors of cancer ( Table 2).

**Table 2.** Suggestions about what should be done for survivors of cancer.

• Symptoms not viewed in isolation but rather as part of a cluster of interrelated problems • Psychosocial interventions to be evidence-based and where possible tailored to the needs of the individual cancer survivor • More effort devoted to disseminating and implementing interventions in practice and to evaluating their cost- effectiveness • Greater attention paid to the needs of vulnerable and high-risk populations of survivors, including the socioeconomically disadvantaged and the elderly

Suggestions collated from Aaronson *et al.* ^26^

## Consequences of psychological distress and disorders

**D**istress symptoms and psychological disorders secondary to cancer have also been shown to have significant negative consequences for both the patient and the family. Maladaptive coping and abnormal illness behaviour have been associated with psychiatric conditions, with negative effects on adherence to treatment, health behaviour, and quality of life ^3^. Also, an increased length of stay in the hospital and/or an increased time in rehabilitation have been found to be more common among patients showing psychiatric symptoms, especially depression, than those with normal adjustment to illness ^11^. Data exist about the reduced response to chemotherapy among depressed breast cancer patients, indicating a possible relationship between psychological disorders and higher risk of recurrence and decreased overall survival ^27^. An increased risk of suicide has also been associated with psychological disorders in cancer ^28^.

Emotional problems such as depression and anxiety, may also reverberate within the family, increasing emotional distress among the patient’s care-givers and, in the case of a patient’s death, risking a greater chance of complicated or traumatic grief among relatives.

In spite of these findings and their implications for clinical care, psychosocial problems in cancer are still minimised and underestimated. Cancer care professionals tend to confuse clinical depression (feeling hopeless, helpless, worthless, or suicidal) or anxiety disorders (phobic avoidance, agitation, constant worry) with normal sadness and preoccupation, with the mistaken belief that “it is normal to feel sad or anxious because of cancer”. The result is that the psychological issues experienced by 30–40% of cancer patients are not identified by the clinician, so these patients are not referred to the correct psychiatric or psycho-oncology service for specialised evaluation and therapy ^29^. However, without better appreciation for and shifts in social attitude towards mental health applied to medicine, including oncology, cancer patients with maladjustment disorders may not receive or accept referral to psycho-oncology services to be assessed and treated for these psychological problems.

## Evaluation and management of psychological and psychosocial dimensions in cancer

In fact, screening and assessment of the emotional problems and concerns of both the patient and the family should in fact be part of every clinical intervention by oncologists and cancer care professionals.

A first step to meet the patient’s needs is the screening of the psychosocial dimensions of cancer as a routine practice in clinical care. The standard application of short clinical tools can be of help in improving the detection of maladjustment and psychosocial or psychological morbidity and referral for care of patients who turn out to be in need of psychological help. The use of some of these instruments, such as the Distress Thermometer or the Edmonton Symptom Assessment System (ESAS), which ask the subject to rate his/her level of symptoms on a visual analogue scale from 0 to 10 and to indicate possible problems or needs, can be helpful for these purposes ^30^. However, the literature also suggests that they cannot be used alone to diagnose depression, anxiety, or distress in cancer patients ^31– 33^. Short or standard tools seem to be more adequate having screening properties enabling them to rule out those who do not have a diagnosis but they lack case-finding properties necessary to confirm clinical caseness ^34^. Some other tools such as the Hospital and Anxiety Depression Scale (HADS) and the Brief Symptom Inventory (BSI) have also been shown to be helpful as a practical system to monitor psychological states across the disease trajectory. Again ^35^, it has to be said that the screening performance of some of these scales (e.g. the HADS-D) is limited compared with standardised psychiatric diagnostic interviews that, in turn, are limited in not being able to identify other psychosocial dimensions of cancer-related suffering, such as demoralisation, health anxiety, and abnormal illness behaviour ^36^.

Screening should be part of the application of the standard of care and clinical practice guidelines, many of which have been developed over the last few years and have been found to be extremely important in helping clinicians provide comprehensive care for cancer patients and their families. The National Comprehensive Cancer Network (NCCN) in the United States has established clinical practice guidelines for the management of distress in oncology that are an example of a specific tool for the routine assessment of psychosocial morbidity and an algorithm for the management of psychological disorders (e.g. adjustment disorders, depression, suicide and suicide risk, and cognitive disorders) in cancer patients. The NCCN screening tool also checks for possible problems in other related areas, including physical, emotional, spiritual, family, and practical problems ^37^. The Canadian National Standards for Psychosocial Oncology (www.capo.ca) also include a series of organisational and educational standards of care and algorithms for the management of anxiety and depression in cancer settings in all phases of the cancer trajectory ^38^, including prevention and survivorship ^39, 40^. The Australian clinical practice guidelines for the psychosocial care of adults with cancer (www.nhmrc.gov.au) published by the National Breast Cancer Centre and the National Cancer Control Initiative are a further example of the work done in this respect to improve the psychological care of cancer patients. These comprehensive guidelines, which have been endorsed by several cancer societies, such as the American College of Surgeons (ACoS), the Commission on Cancer (CoC) ^41^, and the American Society of Clinical Oncology (ASCO) ^42^, as well as other professional societies, including the Association of Oncology Social Work (AOSW) and the Oncology Nursing Society (ONS) ^43^, are gradually transforming the approach to psychosocial care in cancer patients.

Some steps are necessary in order to increase the likelihood that the guidelines are regularly implemented and that distress screening treatment algorithms can be applied ^44, 45^. With respect to this, it is important that, after the screening phase, a more specific evaluation should follow, including a thorough exploration of psychological and behavioural symptoms; this should include previous behavioural health problems, suicidal thoughts, medication use, reliance on substances, and body image and sexuality concerns. Since outcomes do not improve when patients are screened without an established triage algorithm ^46, 47^, cancer care professionals must offer triage to suitable referrals for suffering patients according to their psychosocial needs; these include mental health, social work and counselling, and chaplaincy services. Follow-up with the more distressed patients who require regular and timely assessment based on the established criteria of the screening instruments is a further step in care, and all of these steps and related data (e.g. type, source, and severity of the distress; relevant history; any suicidal ideation; and types of recommended interventions, including a plan for further evaluation and by whom) should be documented in the patient’s treatment record ^48^.

In spite of the dissemination of psychosocial care guidelines, unfortunately it has been reported that, in some areas, such as palliative medicine, healthcare professionals tend to still only partially endorse spiritual and cultural assessment and management and psychosocial assessment and management as important priorities for high-quality services ^49^. Therefore, although some signs of improvement are becoming apparent, a great deal of work is still necessary, especially, but not exclusively, in some parts of the world ^50^.

## Evidence-based psychosocial intervention

Knowledge about the treatment of psychological problems is mandatory for cancer care clinicians, given the number of studies that have shown the efficacy and effectiveness of psychosocial interventions. Counselling, education, coping and psychological support, and more specific forms of psychotherapy in their different formats (group, individual, and family therapy) and orientation (cognitive-behavioural, supportive-expressive, existential, and psychodynamic) have been developed for cancer patients in order to more specifically intervene in all of the conditions where psychological disorders and maladjustment to cancer and treatment emerge. The choice of intervention is related to several variables, including the clinical psychological condition, the type and phase of illness, and the context as well as the availability of psycho-oncology services with trained professionals, which should be part of multidisciplinary teams ^51, 52^.

The literature on the efficacy of the several forms of specialised psychotherapeutic interventions and psychosocial rehabilitation in oncology indicates a general benefit in reducing the severity of psychiatric symptoms ^53^ as well as somatic symptoms (e.g. pain) ^54^ and in improving quality of life, wellbeing, return to work ^55, 56^, illness behaviour, and possibly survival ^57– 59^. The interventions with the most empirical support for treating distress in cancer patients include supportive-expressive group psychotherapy ^60^, cognitive-behavioural ^61^ and cognitive-existential therapy ^62^, meaning-centred psychotherapy ^63, 64^, mindfulness, and mindfulness stress reduction programs ^65– 67^ (see also 68– 70). Also, there is a growing body of evidence supporting the use of integrative therapies, especially mind-body therapies, as effective supportive care strategies during cancer treatment, although many practices remain understudied, with insufficient evidence to be definitively recommended or avoided ^71^.

Psychopharmacological intervention has also been shown to be efficacious in several psychological disorders, where the use of some medications acting on the serotonergic and noradrenergic system (e.g. Selective serotonin reuptake inhibitors and Selective noradrenergic and serotonin reuptake inhibitors) have been shown to help in treating depression and anxiety and cancer-related symptoms, such as hot flashes and pain ^72, 73^. With respect to this, it is important for clinicians, usually psychiatrists but also oncologists and primary care physicians, to have proper training on the use of the drugs, their side effects, and their interactions with other cancer treatment in oncology ^74, 75^.

## Conclusions

Cancer and its treatment have a tremendous psychological and psychosocial impact on both patients and their families and are accompanied by a series of dramatic changes that involve the physical, emotional, spiritual, interpersonal, and social dimensions of the person with cancer. Acknowledging that a high percentage of cancer patients suffer from emotional symptoms (e.g. health anxiety, irritable mood, and demoralisation) or psychopathological conditions (e.g. major depression and post-traumatic stress disorder) is extremely important for cancer care professionals to provide integrated and comprehensive care in oncology. There is now, in fact, scientific evidence of the benefits of providing psychosocial cancer care to patients and families as part of standard care in reducing distress and psychosocial morbidity associated with cancer and in fostering a better quality of life during and after treatment, and eventually increased survival ^76^.

The significant advances in research in the area of psycho-oncology have favoured the development, implementation, and dissemination of psychosocial guidelines and evidence-based treatments for several co-morbid psychiatric disorders in cancer, such as depression ^77^ and anxiety ^78^. However, more steps should be taken in psychosocial approaches to cancer, both in the hospital and in the community ^79^. Besides specific cancer care fields, such as oncology and palliative care, primary care should be particularly considered in its role of continuous, coordinated, and comprehensive care for individuals and families, including psychosocial care, in prevention and diagnosis, in shared follow-up and survivorship care, and in end-of-life care ^80^.
